# Trends in Inpatient Utilization of Head Computerized Tomography Scans in the United States: A Brief Cross-Sectional Study

**DOI:** 10.7759/cureus.26018

**Published:** 2022-06-16

**Authors:** Ali Seifi, Seyedmohammad Jafari, Seyyedmohammadsadeq Mirmoeeni, Amirhossein Azari Jafari, Niyousha Naderi, Armin Safdarpour, Sepehr Seifi

**Affiliations:** 1 Department of Neurosurgery, University of Texas Health Science Center at San Antonio, San Antonio, USA; 2 Life Sciences, McMaster University, Toronto, CAN; 3 Medical School, Shahroud University of Medical Sciences, Shahroud, IRN; 4 Medical School, University of Southern California Keck School of Medicine, Los Angeles, USA; 5 Global Medicine, University of Southern California Keck School of Medicine, Los Angeles, USA; 6 Biomedical Sciences, Texas A&M University, College Station, USA

**Keywords:** hcup-nis, healthcare utilization, imaging utilization trend, head ct, computerized tomography scan

## Abstract

Background

Although computed tomography (CT) has revolutionized the field of medicine due to its incredible diagnostic capabilities, the trends regarding the usage of CT scans, especially in the field of neuroscience, are not very clear. We aim to find the trends in the usage of inpatient head CT scans in the United States using a robust database.

Methods

We queried the national inpatient usage of head CT scans in the United States from 1997 to 2014 using a robust national database. The trends in usage were analyzed based on age, gender, insurance types, and patients’ income.

Results

During the study period, we recorded a total of 5,309,329 head CT scans, of which 51% were female. The total number of head CT scans in the United States dropped significantly from 527,026 cases to 181,095 cases (p=0.000). The decrease was with a steep slope from 1997 to 2002, and since then the decreasing slope turned to a steady state. The decrease in head CT scans was significant in all age groups (p = 0.001), more significant in uninsured payers (-79.4%, p=0.000), and prominent in low-income patients (-70.5 %, p=0.000).

Conclusions

Our study showed that national inpatient usage of CT scans of the head significantly decreased during the past two decades. This decrease is presumably multifactorial: reducing the number of unnecessary radiations, increased appropriateness audits by the government, payers’ payment reductions, and integrated electronic platforms.

## Introduction

Computed tomography (CT) was first introduced in 1972 and this imaging modality and its future derivatives have been an advantage to medical care, especially in neurosurgical conditions such as stroke and intracerebral hemorrhage [1-4].

However, given the cost and level of CT radiation exposure, it has become a major point of concern, especially for clinicians and health policymakers. Effects of low-dose radiation on humans’ health are still controversial, some experts such as Brenner [5] are concerned about the risk of cancer due to radiation with effective doses as low as 10mSv and they proposed that about 2% of cancers could be attributed to radiation from CT scans.

Nowadays, as the optimization of imaging modalities is receiving a lot of attention, especially from commercial payers and Medicare, several measures are taken to reduce the frequency of unnecessary CT scans by health providers [6]. Ironically, despite the increase in the incidence of head injuries, stroke, and cerebral pathologies, there are no large studies that have determined the trends in the utilization of head CT scans.

Bearing the above nuances in mind, in this study we examined the trends of in-patient head CT scan utilization in the United States.

## Materials and methods

Study design, participants, and variables

In this cross-sectional study, Clinical Classifications Software (CCS) was used to query the head CT scans from 1997 to 2014, which is the latest available current data on Healthcare Cost and Utilization Project network (HCUPnet) [7]. All hospital discharges which had head CT scans during the admission were included and there were no exclusion criteria. CCS is freely available and supported by the Healthcare Cost and Utilization Project (HCUP) which is a Federal-State-Industry partnership sponsored by the Agency for Healthcare Research and Quality (AHRQ) [7]. HCUP contains over 7 million inpatients stay information annually and reflects discharges (i.e., the hospital stay) from the United States community hospitals including short-term, non-Federal, general and other specialty hospitals excluding hospital units of other institutions, long-term care facilities such as rehabilitation, psychiatric, and alcoholism, and chemical dependency hospitals [7]. HCUP database represents 97% of the United States population and is the largest available all-payer inpatient care database in the country [7]. This robust database which is the largest healthcare database in the world, reports the patient discharges and if a patient is discharged from the hospital more than once per year it will be counted each time. In the HCUP database, every discharge of any individual per year is counted. Moreover, the year-wise distribution of the number and rate of patients who underwent head CT scanning was included. Outcome variables include their age groups based on the HCUP general age categorization ( <1 year, 1-17, 18-44, 45-64, 65-84, 85+ ), sex, insurance payer, and income status were analyzed in the cohort [7].

Statistical analysis

The relationship of multiple factors such as patients' age, gender, payer, and income with head CT scan usage was evaluated using the Z-test statistical test. Weighted national estimates from HCUP are based on data collected by individual states and provided to AHRQ by the states. In all statistical analyses, a p-value less than 0.001 was considered significant and the estimates of standard errors in HCUPnet were calculated using SUDAAN v. 8.0 software (SAS Institute Inc., Cary, NC) [7]. This study was exempted from full review by the Institutional Review Board of the University of Texas Health at San Antonio (HSC20150408N).

## Results

Utilization of in-patient head CT scans

**Figure 1:**
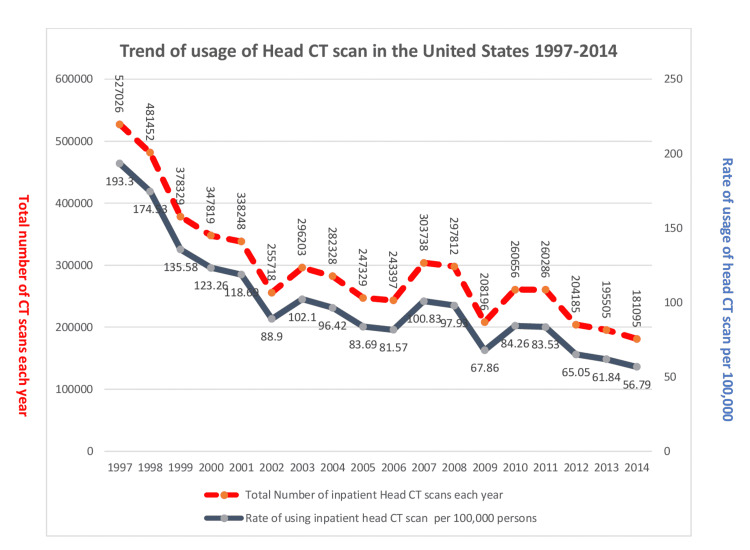
Trend of usage of head CT scan in the United States 1997-2014 The left axis shows the total number of head CT scan usage each year and the axis on the right shows the rate of usage of head CT scans in 100,000 patients/discharges.

During the study period, a total of 5,309,329 discharges with head CT scans were reported, with 51% female patients (Table 1). The total number of in-patient head CT scans per year decreased considerably from 527,026 to 181,095 (p=0.000), with a mean usage of 294,962.71 (+/- 3982) per year. The rate of usage in 100,000 persons, decreased from 193.30 to 56.79 by 70.6% (p=0.000), with a mean rate of 100.90 (+/- 13.48) in 100,000 persons per year. Between 1997 and 2002, the decrease had a sharp slope, but since then, the slope has leveled out (Figure 1).

**Table 1:** Total number of inpatient head CT scans for each year and rate of using one per 100,000 persons

	Total Number of inpatient Head CT scans (Total 5,309,329)	Rate of using inpatient head CT scans (100,000 persons)	p-value
1997	527026	193.3	0.00000
1998	481452	174.53	0.00000
1999	378329	135.58	0.00000
2000	347819	123.26	0.00000
2001	338248	118.69	0.00000
2002	255718	88.9	0.00000
2003	296203	102.1	0.00000
2004	282328	96.42	0.00000
2005	247329	83.69	0.00000
2006	243397	81.57	0.00000
2007	303738	100.83	0.00000
2008	297812	97.93	0.00000
2009	208196	67.86	0.00000
2010	260656	84.26	0.00000
2011	260286	83.53	0.00000
2012	204185	65.05	0.00000
2013	195505	61.84	0.00000
2014	181095	56.79	0.00000

Utilization of in-patient head CT scans based on age range and sex 

The reduction was meaningful across all age groups (P=0.000), and particularly notable in infants (-89.7%), followed by children and teenagers (-85.6%). The frequency of head CT scans was reduced by the same amount in both males (-74.1%) and females (-74.8%), and the decrease was meaningful in both groups (p=0.000) (Table 2).

**Table 2:** Percent changes in the usage of head CT scans during the study period, based on age groups, sex, payer, and income.

	% Change during the Study period	Total Number of inpatient Head CT scans in 1997	Total Number of inpatient Head CT scans in 2014
Age groups (year)			
<1	-89.66	290	32
1-17	-85.62	45	6
18-44	-77.70	83	20
45-64	338248	190	62
65-84	-70.99	720	172
85+	-79.67	1734	497
Sex			
Males	-74.07	257,388 (192.8/100,000)	88,385 (56.3/100,000)
Females	-74.81	269,595 (193.7/100,000)	92,705 (57.3/100,000)
Payer			
Medicare	-62.29	271,484	102,365
Medicaid	-52.99	65,508	30,795
Private	-72.09	132,353	36,940
Uninsured	-79.44	35,048	7,205
Other	-83.40	22,441	3,725
Income			
Low	-70.54	137,979	40,650
Not Low	-62.83	356,970	132,685
Missing	-75.81	32,077	7,760

Utilization of in-patient head CT scans based on payer and income

Based on Table 2, the analysis of the insurance types showed that although all types of insurance significantly had a lower usage of head CT scans in the recent years, the decline was more pronounced among uninsured patients (-79.4%, p=0.000) in comparison to payers with private insurance (-72.1%, p=0.000), Medicare (-62.3%, p=0.000), and Medicaid (-53.0%, p=0.000). Finally, the usage of head CT decreased at all income levels; however, the decline was more significant in low-income patients (-70.5%) compared to others (-62.8%) (p<0.001).

## Discussion

Our study showed that the usage of inpatient head CT usage has been decreasing in the USA during the study period. To the best of our knowledge, this is the first published article on the HCUP database that analyzes the trend of usage of head CT in the United States. Although in the past several years, it has been reported that there is a flattening in the growth of the referral patients for CT, according to our results, the use of head CT scans has declined significantly during the last nearly two decades [6]. It could be due to several factors, such as increased awareness of potential radiation risks caused hesitancy in requesting and performing CT scans unless in necessary situations [8]. Also, the efforts by organizations, for instance, the Food and Drug Administration (FDA), the American College of Radiology (ACR), as well as General Electric (GE) healthcare company to reduce the number of excessive radiations could be a contributor [9,10]. In addition, what we have found on the basis of prior studies is that there was a notable reduction of head CT scans in infants, followed by children and teenagers [11]. Worldwide, the CT usage in adults continues to slow down than seen previously, likely due to the awareness of ionizing radiation exposure. Furthermore, the decline of CT usage in children and a more significant increase in MRI may reflect greater awareness of the concern regarding radiation exposure and harm in children [11]. The authors hypothesize that the observed drop in the usage of head CT scan usage may be associated with more brain MRI availability recently as compared to 20 years ago, and of course with more anatomical head and neck details and fewer side effects. 

Another possible reason for our finding of a decrease in the usage of head CT scans in recent years could be more restricted insurance rules and reimbursement limitations. Alarmingly, it is revealed that dropping in the utilization rate of CT by 1.7% in Medicare patients in 2010 whether is beneficial or detrimental to the healthcare system is coincident with our findings [6]. Given the fact that based on our results the desire to use head CT has been reduced in all income levels, particularly the low-income compared to others, variant strategies are applied to reduce the financial burden and enhance CT access [12]. Recently, efforts made by payers such as Medicare to minimize the unnecessary procedures and cost of health care services could be other factors [6]. One main reason is the healthcare leadership culture change that started in the early 21st century and advocated for increased appropriateness in any type of testing. The possible overuse of diagnostic testing has been addressed by payers and several medical boards’ campaigns to reduce imaging through payment reductions [11]. In the authors' institution, the usage of CT scans and the repeated scans during the admission is being discussed during the bedside rounds to make sure performing the new scan is really needed and can add value or contribute to the plan of care. Especially in the teaching institutions, one of our main goals as healthcare educators should be teaching our in-training healthcare professionals to think before any lab or radiology request. Residents and medical students should evaluate the cost-benefits of each procedure and discuss whether this extra workup can change the patient's treatment plan, and not just do it for their own curiosity. 

Lastly, the other hypothetical reason for the lower usage of CT scans toward the end of the study period, in our opinion, is the emerging computer technology and sharing cloud storage in recent years. Several years ago, the CT scans used to be printed on the radiology films, and the patient used to carry them and if they missed it, they had to repeat the CT scans. But nowadays thanks to the cloud storage systems, the physicians can reach out to the patients to scan through the cloud, even if the patient has lost the radiology film or the disc. Utilization of a centralized platform and integration of Clinical Decision Support Systems (CDSS) and Picture Archiving and Communication Systems (PACS) in hospitals are important measures that lessen the probability of duplicate imaging and assist physicians in making evidence-based decisions in a timely fashion [13].

Our study had several limitations, despite our data being queried on a robust national database. HCUP doesn't provide detailed information about the reasons for the CT scans that have been performed, so we can not comment if the CT usage has been unnecessary. Also, analysis of the HCUP database can not lead to a casualty but shows only an association. Being said we had limitations in finding the exact causes of decreases in the usage of head CT scans in the past two decades, and we could only assume the reasons behind the associations. 

## Conclusions

In summary, our findings based on a robust national database suggest that while there has been a reduction in the inpatient usage of head CT imaging from 1997 to 2014, there are continued opportunities for appropriate imaging. The decreasing trend of inpatient CT scan usage is possibly multifactorial. Some possible reasons include more awareness about the radiation side effects, insurance reimbursement limitations, better healthcare leadership management, and lastly emerging digital technologies and storage. Clinicians should be aware of the potential side effects of radiation; however, the payer types and patient disparities should not impact the decision for the necessary testing and imaging.
